# Effects of Combinations of Ophthalmic Viscosurgical Devices and Suction Flow Rates on the Corneal Endothelial Cell Damage Incurred during Phacoemulsification

**DOI:** 10.1155/2020/2159363

**Published:** 2020-07-21

**Authors:** Tomoyuki Kunishige, Hiroshi Takahashi

**Affiliations:** Department of Ophthalmology, Nippon Medical School, 1-1-5 Sendagi, Bunkyo-ku, Tokyo 113-8603, Japan

## Abstract

We examined the effects of different ophthalmic viscosurgical devices (OVDs) and suction flow rates during phacoemulsification on the amount of ultrasound power used and damage to the corneal endothelium. In total, 48 eyes of 24 patients who underwent phacoemulsification and intraocular lens insertion with different OVD settings in the left and right eye between February and August 2018 were examined retrospectively from medical records. Each of the following types of OVDs was used in either the right or left eye of each patient: a viscoadaptive OVD (V group) or a combination of dispersive and cohesive OVDs (soft-shell technique; S group). There was no significant difference in the lens nucleus hardness between the two groups. A 2.4 mm transconjunctival scleral incision was made, and phacoemulsification was performed by the same surgeon. The cumulative dissipated energy (CDE) and ultrasound time intraoperatively were compared between the two groups. The CDE was significantly larger in the V group (9.9 ± 4.6) than the S group (6.4 ± 3.0; *p*=0.006). The reduction rate of the endothelial cell density at the center of the cornea was significantly higher in the V group (4.1% ± 6.7%) than the S group (0.3% ± 4.5%; *p*=0.03) at 1 week postoperatively. Both groups had a good postoperative course. There was less corneal endothelial damage with the soft-shell technique combined with a normal flow setting than the viscoadaptive OVD combined with a low flow setting.

## 1. Introduction

Although the safety of phacoemulsification cataract surgery has been fairly well established, surgical complications, such as corneal endothelial cell damage, have not yet been fully overcome [1–6]. Several studies compared the complications which were associated with femtosecond laser-assisted cataract surgery (FLACS) versus the conventional phacoemulsification surgery and showed that FLACS did not improve intra/postoperative complications in comparison to the conventional phacoemulsification surgery, and it cannot be considered cost-effective [7, 8]. Therefore, the choice of OVDs and the settings of the phacoemulsification machine are important factors in preventing corneal endothelial damage [9, 10]. Various OVDs have been developed to protect corneal endothelial cells. During phacoemulsification, it is important to maintain OVDs in the anterior chamber to protect corneal endothelial cells. For this purpose, the settings for the suction flow rate and pressure and the OVD behavior are important [10]. Each OVD exhibits a specific movement in the anterior chamber that depends on the irrigation/suction flow setting. Dispersive OVDs tend to remain in the anterior chamber, while cohesive OVDs easily disappear under a normal setting. In this regard, the soft-shell technique, i.e., the combined application of a dispersive OVD, which is injected into the corneal endothelium side, and a cohesive OVD, which is filled in the anterior chamber, appears to be useful in protecting the corneal endothelium [11]. However, the behavior of viscoadaptive OVDs changes greatly depending on the setting of the suction flow rate: with a low flow rate (less than about 25 cc/min), it remains in the anterior chamber, and with a high flow rate, it can be easily aspirated and removed [12, 13]. The relationship between the choice of OVDs and corneal endothelial damage has been discussed in various papers [14–18], and a meta-analysis review showed that viscoadaptive OVDs and the soft-shell technique are superior to other OVDs [19]. However, there have been few reports comparing them under different suction flow settings. In this study, we compared the corneal endothelial damage incurred during phacoemulsification using a viscoadaptive OVD with a low flow setting and a soft-shell OVD technique with a normal flow setting.

## 2. Materials and Methods

This study adhered to the tenets of the Declaration of Helsinki and was approved by the Institutional Review Board/Ethics Committee of Nippon Medical School Hospital (no. R1-06-1155). We followed the retrospective observational research information disclosure procedure (opt-out) of Nippon Medical School Hospital to obtain informed consent from the research participants. In total, 48 eyes of 24 patients who underwent phacoemulsification and intraocular lens insertion with different OVD settings in the left and right eye at Nippon Medical School Hospital between February 2018 and August 2018 were examined retrospectively from medical records. A viscoadaptive OVD (Healon V®, AMO Japan K.K., Tokyo, Japan; the V group) or the soft-shell technique (combination of a dispersive OVD, Shellgan®, and a cohesive OVD, Opegan Hi®, Santen Pharmaceutical Co., Osaka, Japan; the S group) was used in the right or left eye. Assignment of the choice of OVDs was determined nonintentionally. All surgeries were performed by a single surgeon (T.K.). The grade of the lens nucleus hardness was evaluated preoperatively according to the LOC III classification [20, 21].

A 2.4 mm transconjunctival scleral incision was made, and phacoemulsification was performed using standardized techniques with CENTURION® Vision System (Alcon Japan Ltd., Tokyo, Japan) under the torsional ultrasound oscillation mode. During phacoemulsification, the suction flow rate was set to 18 mL/min in the V group and 35 mL/min in the S group to obtain good retention of each OVD in the anterior chamber. The vacuum pressure was set to 200 mmHg in the V group and 340 mmHg in the S group, and the intraocular pressure (IOP) was set to 34 mmHg in the V group and 40 mmHg in the S group. These different settings in each group were applied to maintain a proper anterior chamber depth. Ultrasound power levels were set to 40% in both groups. The cumulative dissipated energy (CDE) and ultrasound time were recorded intraoperatively. The CDE is the mean of the ultrasound power used intraoperatively. In this study, it was calculated as follows: CDE = torsional amplitude × torsional time × 0.4. The number 0.4 is empirically used for standardization of the torsional oscillation to allow comparison with the conventional longitudinal oscillation [22]. All parameters were automatically calculated by the machine.

The rate of reduction of the corneal endothelial cell density (ECD) at the center of the cornea was assessed by noncontact specular microscopy (Noncon Robo, Konan, Hyogo, Japan). The measurement was performed preoperatively and at 1 day, 1 week, and 1 month postoperatively. At the same time points, the central corneal thickness (CCT) and the central 10.0 mm corneal volume (CV) were also evaluated by Pentacam (Oculus Optikgeräte GmbH, Wetzlar, Germany).

The CDE, ultrasound time, ECD reduction rate, CCT, and CV in both groups were compared using the Mann–Whitney U test. Probability (*p*) values < 0.05 were considered to be statistically significant.

## 3. Results

The data of 48 eyes of 24 patients were retrospectively analyzed. The patients ranged from 65 to 89 years of age. Preoperatively, there was no significant difference between the V group and the S group in the lens nucleus hardness as evaluated by the LOC III classification, ECD, CCT, and CV (Table 1).

**Figure 1:**
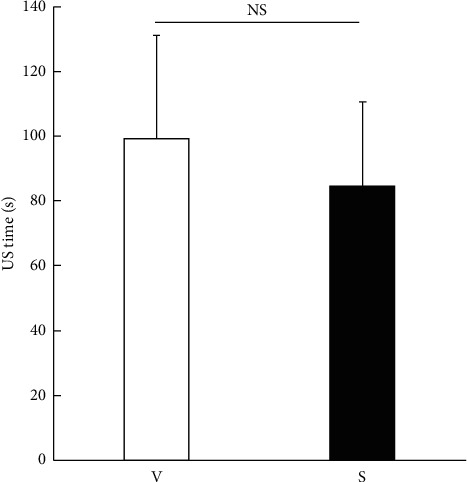
Comparison of ultrasound (US) time. The intraoperative ultrasound time is compared between the two groups with no significant difference (Mann–Whitney U test; *p*=0.11). NS = not significant, V = V group, and S = S group.

**Figure 2:**
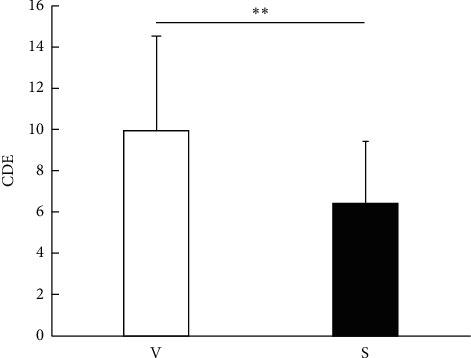
Comparison of the cumulative dissipated energy (CDE). The intraoperative CDE is compared between the two groups. It is significantly higher in the V group than the S group (*p*=0.006; Mann–Whitney U test; ^*∗∗*^*p* < 0.01). V = V group and S = S group.

**Figure 3:**
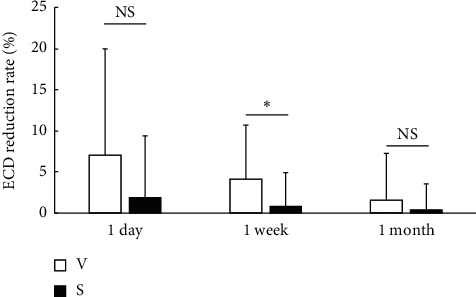
Comparison of the corneal endothelial cell density (ECD) reduction rate. The ECD reduction rate (mean ± standard deviation) is calculated from the preoperative ECD and the ECD at each time point. ECD rate is significantly higher in the V group than the S group at 1 week postoperatively (*p*=0.13 at 1 day, *p*=0.03 at 1 week, and *p*=0.43 at 1 month; Mann–Whitney U test; ^*∗*^*p* < 0.05). NS = not significant, V = V group, and S = S group.

All surgeries were performed without any serious intraoperative complications, such as posterior capsule rupture, vitreous loss, or iatrogenic zonular dialysis. The ultrasound time did not differ significantly between the two groups (99.4 ± 31.8 s in the V group and 84.7 ± 26.1 s in the S group; Figure 1), while the CDE was significantly higher in the V group (9.9 ± 4.6) than the S group (6.4 ± 3.0; *p*=0.006; Figure 2). The ECD reduction rate did not differ significantly between the two groups at 1 day and 1 month postoperatively; however, the ECD reduction rate at 1 week postoperatively was significantly higher in the V group (4.1% ± 6.7%) than the S group (0.3% ± 4.5%; *p*=0.03; Figure 3). The changes in the CCT and CV did not differ significantly between the two groups at any time point (Table 2).

## 4. Discussion

In the current study, there was no significant difference in the ultrasound time between the V group and the S group, while the ultrasound energy used in surgery, i.e., the CDE, was significantly higher in the V group than the S group. A characteristic behavior of viscoadaptive OVDs in the anterior chamber, i.e., its tendency to remain in place when a low suction flow rate is used and to be removed easily when a high suction flow rate is used [12, 13], may have caused this difference. When the anterior chamber is fully filled by a viscoadaptive OVD, extra ultrasound oscillation may be necessary to emulsify and aspirate the lens nuclei, which may have contributed to the larger ECD reduction rate in the V group when compared with the S group at 1 week postoperatively.

In ophthalmic surgery, various methods for assessing surgical invasion have been reported [23, 24]. We observed the behavior of OVDs during phacoemulsification using the “slit side view” method and fluorescein-stained OVDs, which enabled viewing of the movement of OVDs intraoperatively and the fluid dynamics in the anterior chamber [25]. With the soft-shell technique, the dispersive OVD formed an uneven layer on the surface of the corneal endothelium, where it remained during the phacoemulsification procedure. In contrast, the viscoadaptive OVD remained inside the anterior chamber as a lump, and the irrigation solution often flowed between the corneal endothelium and the OVD, leading to the detachment of the OVD [25]. These mechanisms may have contributed to the larger ECD reduction rate seen in the V group when compared with the S group at 1 week postoperatively.

The ease of use of different types of OVDs is one of the most important factors for the success of cataract surgery, especially for medical residents. Tetz et al. reported that surgeons graded viscoadaptive OVDs to have a better overall surgical performance and retention in the anterior chamber during phacoemulsification than dispersive OVDs [17]. Phacoemulsification with a low suction flow rate and leaving the viscoadaptive OVD in place until the end of the procedure seem effective for residents in protecting the corneal endothelium. In addition, phacoemulsification with a low suction flow rate reduces the risk of complications, such as posterior capsule rupture or vitreous prolapse. Thus, viscoadaptive OVDs are useful when used with the knowledge of their characteristic behavior in the anterior chamber.

In interpreting the results of this study, several limitations need to be considered. This study was small and retrospective in nature. In addition, cases with very shallow anterior chamber depths, very hard nuclei, or very weak zonules and complex cases, such as those requiring high ultrasound power or long operating times, were not included.

## 5. Conclusions

There was less corneal endothelial damage from phacoemulsification with the soft-shell technique combined with a normal suction flow setting than with the viscoadaptive OVD combined with a low suction flow setting. Further studies are needed to prove this hypothesis.

## Figures and Tables

**Table 1:** Preoperative evaluations.

	V group, mean (SD)	S group, mean (SD)	*p* value
Nucleus hardness, NC, LOC III	3.1 ± 0.5	3.1 ± 0.6	0.53
ECD (cells/mm^2^)	2914.9 ± 240.1	2850.3 ± 193.7	0.31
CCT (*μ*m)	555.5 ± 36.6	554.0 ± 38.7	0.53
CV (mm^3^)	60.6 ± 3.0	60.8 ± 3.3	0.53

NC = nucleus color; LOC III = Lens Opacities Classification System III; ECD = endothelial cell density; CCT = central corneal thickness; CV = 10.0 mm corneal volume; and SD = standard deviation.

**Table 2:** The rate of increase in CCT and central 10.0 mm CV.

	V group, mean (SD)	S group, mean (SD)	*p* value
*The rate of increase in CCT, %*			
1 day after surgery	3.38 ± 3.74	3.47 ± 3.55	0.55
1 week after surgery	2.20 ± 3.56	2.40 ± 2.89	0.47
1 month after surgery	0.98 ± 3.23	0.74 ± 2.29	0.54

*The rate of increase in CV, %*			
1 day after surgery	5.66 ± 3.55	6.94 ± 3.86	0.39
1 week after surgery	4.05 ± 3.32	5.13 ± 3.18	0.27
1 month after surgery	1.49 ± 2.25	1.08 ± 3.47	0.55

CCT = central corneal thickness; CV = 10.0 mm corneal volume; and SD = standard deviation.

## Data Availability

The data used to support the findings of this study are included within the article.
